# Molecular detection of *Setaria tundra *(Nematoda: Filarioidea) and an unidentified filarial species in mosquitoes in Germany

**DOI:** 10.1186/1756-3305-5-14

**Published:** 2012-01-11

**Authors:** Christina Czajka, Norbert Becker, Sven Poppert, Hanna Jöst, Jonas Schmidt-Chanasit, Andreas Krüger

**Affiliations:** 1 German Mosquito Control Association (KABS), Waldsee, Germany; 2 University of Heidelberg, Heidelberg, Germany; 3 Department of Parasitology, Bernhard Nocht Institute for Tropical Medicine, Hamburg, Germany; 4 Department of Virology, Bernhard Nocht Institute for Tropical Medicine, Hamburg, Germany; 5 Department of Tropical Medicine, Bundeswehr Hospital Hamburg, Hamburg, Germany

**Keywords:** Culicidae, filariae, *Setaria tundra*, mtDNA marker, Germany

## Abstract

**Background:**

Knowledge of the potential vector role of Culicidae mosquitoes in Germany is very scanty, and until recently it was generally assumed that they are not involved in the transmission of anthroponotic or zoonotic pathogens in this country. However, anticipated changes in the course of global warming and globalization may alter their status.

**Methods:**

We conducted a molecular mass screening of mosquitoes for filarial parasites using mitochondrial 12S rRNA-based real-time PCR.

**Results:**

No parasites causing disease in humans such as *Dirofilaria *spp. were detected in about 83,000 mosquitoes tested, which had been collected in 2009 and 2010 in 16 locations throughout Germany. However, minimum infection rates of up to 24 per 1000 mosquitoes were revealed, which could be attributed to mosquito infection with *Setaria tundra *and a yet unidentified second parasite. *Setaria tundra *was found to be widespread in southern Germany in various mosquito species, except *Culex *spp. In contrast, the unidentified filarial species was exclusively found in *Culex *spp. in northern Baden-Württemberg, and is likely to be a bird parasite.

**Conclusions:**

Although dirofilariasis appears to be emerging and spreading in Europe, the absence of *Dirofilaria *spp. or other zoonotic filariae in our sample allows the conclusion that the risk of autochthonous infection in Germany is still very low. Potential vectors of *S. tundra *in Germany are *Ochlerotatus sticticus*, *Oc. cantans*, *Aedes vexans *and *Anopheles claviger*. Technically, the synergism between entomologists, virologists and parasitologists, combined with state-of-the-art methods allows a very efficient near-real-time monitoring of a wide spectrum of both human and veterinary pathogens, including new distribution records of parasite species and the incrimination of their potential vectors.

## Background

Filariae (order Spirurida, superfamily Filarioidea) represent a relatively small group of tissue-dwelling, parasitic nematodes with great impact on human and animal health [1]. Those of highest medical relevance are the causative agents of lymphatic filariasis (*Wuchereria bancrofti *and others) and onchocerciasis (*Onchocerca volvulus*), both diseases with anthroponotic cycles. Several other filarioses are truly enzootic, e.g. in ungulates (animal onchocerciasis) or rodents (*Litomosoides *sp.), but a few may be transmissible on rare occasions from animals to humans, such as dirofilariasis [2-4] or accidental onchocerciasis [5,6]. Although dirofilariasis has been diagnosed in Germany both in humans and dogs, it is assumed that most dog cases and all human cases were imported from endemic regions outside Germany [7-9]. However, more recently *Dirofilaria *spp. are considered emerging agents of parasitic zoonoses in Europe with a northward trend of expansion [10].

In general, filarioses are vector-borne infections transmitted by various haematophagous arthropods, e.g. insects such as mosquitoes (Culicidae) or arachnids such as mites (Acari) [11,12]. However, the role of particular vectors in the transmission cycles of many filarial species, and their geographical distribution remain largely unknown. These gaps can partially be attributed to the very laborious screening techniques used until recently. If infectivity, prevalence, and parasite identity were to be determined from a vector, large numbers of the arthropods had to be collected and freshly dissected.

Nowadays, molecular techniques are frequently used to detect filarial species in arthropods [13-16]. They allow a much faster throughput and less specimen handling, which is of particular relevance when the prevalence of the parasite is very low. However, these techniques can only provide information about the presence of particular parasites; they do not allow definite conclusions about the natural vector status or infectivity, unless the different body segments such as heads of the insects are screened separately.

Filarial species (for authority names and dates of filarial species please refer to [12,17]), known in mammals from Germany include *Onchocerca flexuosa*, *O. skrjabini *(syn. *O. tarsicola*) and *O. jakutensis *(all in red deer; [18]), *O. gutturosa *and *O. lienalis *(in cattle; [19]), *Dirofilaria immitis *and *D. repens *(from dogs and humans, but presumably all imported; [9,20]), *Setaria tundra *(in roe-deer; [21]), *Parafilaria bovicola *(in cattle; [22]), and *Cercopithifilaria rugosicauda *(in roe-deer; [23]). Besides these, bird filariae, for example *Cardiofilaria*, *Eufilaria *or *Sarconema *can be expected in Germany [12].

The vectors of the filariae mentioned in Germany are to a large extent unknown. Schulz-Key and Wenk [24] incriminated *Simulium ornatum *and *Prosimulium nigripes *as natural vectors of the red deer parasite *O. tarsicola *(= *O. skrjabini*). Beyond that, it can be assumed from studies in other European countries and in Japan that several *Onchocerca *spp. are transmitted by blackflies and biting midges [12,25,26], the *Dirofilaria *species by various mosquitoes (Italy: [27]), *Eufilaria *spp. by biting midges (France: [12]) and *Setaria tundra *by *Aedes *mosquitoes (Finland: [28]).

In order to (i) further clarify the occurrence of mosquito-borne filariae in Germany, (ii) identify their potential vectors, and (iii) monitor the possible introduction or expansion of parasites due to climate change or globalization, we conducted a mass collection of mosquitoes in 2009 and 2010. Alongside a screening for arboviruses in German mosquitoes [29-31], the same mosquito specimens were co-tested for the presence of filariae. In addition, mosquitoes were tested for filariae that were not included in the arbovirus screening.

## Methods

**Figure 1:**
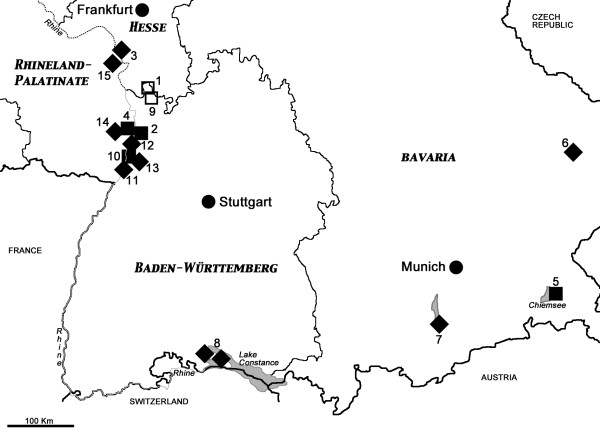
**Locations of the study sites in south-western Germany**. Numbers refer to Table 1 (site 16 not shown on this map). Symbols: diamonds, mosquito collection sites; open squares, positive mosquitoes for unidentified filariae; solid squares, positive mosquitoes for *Setaria tundra*.

All procedures basically followed those used in the previously established arbovirus surveillance program [29]. Mosquitoes were trapped from July to September 2009 and from April to September 2010 at 15 sites in southern Germany and one in eastern Germany (see Table 1 and Figure 1), with CO_2_-baited EVS (encephalitis vector survey) traps (BioQuip, Compton, CA, USA) and with gravid traps (GT) designed according to the CDC gravid trap model 1712 (John W. Hock Company, Gainesville, FL, USA). GT's were used in urban and peridomestic areas, whereas EVS trapping was performed in natural habitats, e.g. wetlands, flood plains, wet woodlands. Mosquitoes collected were frozen at -70°C, transported to the laboratory, and identified on chill tables to species, species complex (*An. maculipennis *s.l.) or genus (*Culex *spp., which stands for *Cx. pipiens *and *Cx. torrentium*), and sex using morphological characteristics [32]. The processing over chill tables, however, could not always be conducted in 2010.

**Table 1:** Collection and infection details of mosquitoes.

**Site No**.	Federal State	Location	Coordinates N/E	Trap type	No. of mosquitoes	No. of pools	Pool sizes	No. of pos. pools	MIR*
	**2009**								

1	**B-W**	Weinheim	49°33'/8°40'	GT	3699	165	1-25	34	9.2

2	**B-W**	Wagbachniederung (Wäghäusel)	49°15'25"/8°31'08"	GT	888	45	1-26	11	12.4
				EVS	7237	336	2-28	18	2.5

3	**R-P**	Kühkopf Knoblochsaue/Flotzengrün	49°49'/8°24'	EVS	2532	134	2-25	0	0
4			49°17'/8°25'	EVS	166	9	1-25	4	24.1

	**Total 2009**				**14522**	**666**	**1-28**	**67**	**4.6**

	**2010**								

5	**Bav**	Chiemsee	47°51'28"/12°31'08"	EVS	16583	680	1-27	1	0.06

6	**Bav**	Isar	48°47'14"/12°55'07"	EVS	3314	137	5-25	0	0

7	**Bav**	Osterseen	47°46'23"/11°18'25"	EVS	8030	331	1-25	0	0

8	**B-W**	Lake Konstanz	47°44'34"/8°58'59"	EVS	10092	442	1-25	1	0.09
			47°44'17"/8°58'52"						
			47°41'40"/9°06'53"						

9	**B-W**	Großsachsen	49°31'/8°40'	GT	3327	175	1-25	99	29.75

10	**B-W**	Karlsruhe-Rott Island	49°09'02"/8°23'10"	EVS	11960	506	1-50	3	0.25

11	**B-W**	Karlsruhe-Knielinger Lake	49°02'04"/8°18'50"	EVS	13	4	1-8	0	0

12	**B-W**	Karlsruhe-Russheim	49°11'26"/8°25'01"	EVS	84	7	2-25	0	0

13	**B-W**	Karlsruhe-Stutensee	49°04'/8°30'	EVS	3	2	1-2	0	0

1	**B-W**	Weinheim	49°33'/8°40'	GT	1607	103	1-25	40	24.89

2	**B-W**	Wagbachniederung (Wäghäusel)	49°15'25"/8°31'08"	EVS	678	41	1-30	4	5.89

14	**R-P**	Haßloch	49°18'/8°17'	EVS	888	48	1-25	0	0

15	**R-P**	Mainz	49°45'/8°18'	EVS	356	21	1-25	0	0

16	**S-A**	Coswig/Elbe	51°51'/12°26'	EVS	11698	484	1-25	1	0.08

	**Total 2010**				**68633**	**2981**	**1-50**	**149**	**2.17**

	**Total 2009 and 2010**				**83155**	**3647**	**1-50**	**216**	**2.6**

For general information on species compositions see [31].

Abbreviations: B-W: Baden-Württemberg; Bav: Bavaria; EVS: Encephalitis vector survey trap; GT: Gravid trap; MIR: minimum infection rate; R-P: Rhineland-Palatinate; S-A: Saxonia-Anhalt. *These Minimum Infection Rates apply to the locality and year only, and include all local mosquito species and both parasite species, respectively. For mosquito species-wise MIR's please refer to the Results section.

As a large number of caught mosquitoes needed to be examined, they were merged into pools. For the further investigation it was not necessary to know exactly which mosquito was infected but which species at which trapping site was positive for filariae. Hence each pool normally consisted of up to 25 individuals from the same species collected at the same trapping site, placed in sterile 2-ml cryovials, and then maintained at -70°C until assayed. As shown in the results, knowing the species is important for the interpretation of the collected data. In the next step, each 5 μl of extracted DNA of five pools and 65 μl of water were merged to super-pools, which were used for the first screen with the filarial-specific real-time PCR (dilution 1:10). The positive super-pools were further examined, by screening each single pool of the super-pool again with the filarial-specific real-time PCR.

Each mosquito pool was triturated in 500 μl of cell culture medium (high-glucose Dulbecco's modified Eagle's medium [DMEM; Sigma-Aldrich, St. Louis, MO, USA] with 10% heat-inactivated foetal bovine serum, 100 U/ml penicillin, 100 μg/ml streptomycin, and 2.5 μg/ml amphotericin B) with two stainless steel beads (5 mm) in a TissueLyser (Qiagen, Hilden, Germany) for 2 min at 50 oscillation/s. The suspensions were clarified by centrifugation (5000 × *g *for 1 min), and the supernatant was used for nucleic acid extraction with a QIAamp viral RNA mini kit according to the manufacturer's protocol (Qiagen, Hilden, Germany).

The filaria-specific real-time PCR, targeting a 94 bp long fragment of the 12S rRNA gene from the mitochondrial genome, was performed using the primers FILA-F (5' TGG ATT AGT ACC CAG GTA ATC 3') and FILA-R (5' CCA AAG AAA AAT CTA AAG TCA GTC 3') and LNA probe FILA-P (5' FAM AAC+AAA+ACT+TTA+CTCCCGA-BHQ1 3' [FAM = 6-carboxyfluorescein; BHQ1 = black hole quencher 1]). Real-time PCR was performed with QuantiFast Probe PCR kit according to the manufacturer's protocol (Qiagen).

Real-time PCR-positive pools were subsequently examined by a conventional PCR (with subsequent gel electrophoresis) targeting an approx. 500 bp long fragment of 12S rRNA (ribosomal RNA), 16S rRNA and *COI *(cytochrome oxidase I gene) [33,34]. The resulting PCR products were purified with the NucleoSpin Extract II kit (Macherey-Nagel, Düren, Germany) and commercially sequenced in both directions using the PCR primers (Seqlab GmbH, Göttingen, Germany) by direct sequencing of PCR products.

**Figure 2:**
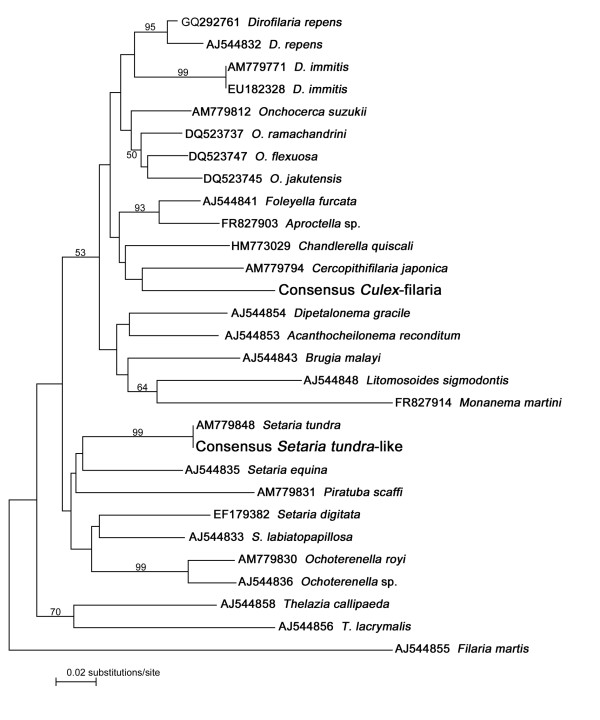
**Classification of the two filariae under study**. Taxon identification tree based on partial 12S rDNA sequences (Neighbour-joining algorithm with Kimura 2-parameter distances). New records are highlighted in large letters. Numbers above branches indicate statistical bootstrap support of ≥ 50%.

The respective sequences of six specimens are deposited in [GenBank: JN228376-JN228381]. Sequences were aligned with ClustalW2 [35], after adding several reference sequences from GenBank. The alignment file was uploaded in MEGA 5.05 [36], which was used to calculate the taxon ID tree (Neighbour-Joining algorithm) with consensus sequences in comparison with previously published species sequences (see Figure 2).

## Results and discussion

A total of 14,522 female mosquitoes were collected in 2009 (Table 1). The collections consisted of *Culex *spp. (confirmed: *Cx. pipiens *and *Cx. torrentium*; not confirmed: *Cx. modestus*, *Cx. territans*), *Aedes vexans*, *Ae. cinereus*, *Ochlerotatus cantans*, *Oc. sticticus*, *Culiseta annulata*, *Anopheles maculipennis *s.l., and *An. claviger*. The most abundant species were *Culex *spp. (35%), *An. claviger *(24%) and the floodwater mosquito *Ae. vexans *(20%). The mosquitoes most frequently trapped with the gravid traps (GT) were *Culex *spp. (99%), while the total catches in GTs were 4,587, compared with 9,935 specimens trapped with the EVS traps (proportion 1:2.2).

Overall, in the first-round real-time PCRs, 218 super-pools, which had been derived from 666 original pools, were assayed for the presence of filariae. Of the 218 super-pools, 63 (29%) gave filaria-positive results.

Of the 63 positive super-pools, all respective original pools (189) were screened by real-time PCR, and 67 (35.4%) were shown to be positive, of which 24 were confirmed by 12S PCR (23 were sequenced). In 17 cases (27%), the second round-PCR did not confirm the positive first-round result.

Of 226 *Culex *spp. pools consisting of 5106 individuals, 44 pools were filaria-positive by real-time PCR, which corresponds to 19.5% positive *Culex *spp. pools and a minimum infection rate (MIR: [number of positive pools/total specimens tested] × 1000) of 8.6 per 1000 mosquitoes.

Of 369 non-*Culex *pools (excluding *Culiseta *and *An. maculipennis *s.l.) consisting of 8538 individuals of *Ae. vexans*, *Oc. cantans*, *Oc. sticticus *and *An. claviger*, 23 were filaria-positive, which corresponds to 6% positive pools and a MIR of 2.7. In terms of species, the MIRs were as follows: *Oc. sticticus *6.2, *An. claviger *2.3, *Ae. vexans *1,7. The highest MIR at a single locality was 24.1 at Flotzengrün (Table 1), which was entirely due to positive *Ae. vexans *pools.

In 2010, a total of 68,633 female mosquitoes were collected and processed into 2981 pools. In addition to the species caught in 2009, *Oc. annulipes*, *Oc. communis*, *Oc. punctor*, *Oc. rusticus *and *An. plumbeus *were caught. *Aedes vexans *was the dominating species (50%), followed by *Oc. sticticus *(35%) and *Culex *spp. (8%). The mosquitoes most frequently trapped with the GT were again *Culex *spp. (> 99%), with a total proportion of GT versus EVS of 1:13.

Of the 2981 pools, 149 were tested filaria-positive by real-time PCR (5%), of which 54 were confirmed by either 12S, or 16S or *COI *PCR, and 27 12S products were sequenced. In 78 cases (52%), the second-round PCR did not confirm the positive first-round result.

Of 307 *Culex *spp. pools consisting of 5417 individuals, 140 pools were filaria-positive by real-time PCR, which corresponds to 45% positive *Culex *spp. pools and a MIR of 25.8.

Of 2674 non-*Culex *pools consisting of 63,216 individuals of 12 different species, only nine were filaria-positive (four *Ae. vexans*, two *An. claviger*, two *Cs. annulata*, one *Oc. sticticus*), which corresponds to 0.3% positive pools and a MIR of 0.1. The highest MIR at a single locality was 29.75 at Großsachsen (Table 1), which was entirely due to positive *Culex *spp. pools.

In 2009, fourteen closely related filarial sequences resembling that of *Setaria tundra *(identity of consensus with *S. tundra *is 97-99%) were derived from five pools of *Oc. sticticus*, four pools each of *Ae. vexans *and *An. claviger*, and one pool of *Oc. cantans*. In 2010, only two additional *S. tundra-*like sequences were revealed from one *Oc. sticticus *pool and one *Ae. vexans *pool. The *S. tundra *sequences (as consensus in Figure 2) unequivocally cluster with other *S. tundra *(99% bootstrap support).

Additionally, in 9 and 25 pools of *Culex *sp. (2009 and 2010, respectively) sequences of unspecified filarial origin were derived, but presumably all of the same species. In the 12S taxon identification tree (Figure 2) these *Culex *filariae (as consensus) do not cluster with any of those included in the tree analysis and cannot even be assigned unequivocally to any of the genera sequenced so far (see also below). An additional analysis with a reduced number of taxa (all those in the branch above *Dipetalonema *in Figure 2) revealed an identical topology, but with higher statistical bootstrap support (data not shown). A BLAST search of GenBank retrieves the highest sequence identity (86%) with *Onchocerca suzukii*, a parasite of Japanese bovids. However, the tree (in Figure 2) shows that this does not correspond to a close relationship, as *O. suzukii *clusters with other *Onchocerca *species.

### Geographic pattern

As the yet unidentified filarial species was exclusively found in *Culex *mosquitoes, it is not surprising that it was most abundant in those places where mainly *Culex *spp. were caught using gravid traps, which were Weinheim and Großsachsen (Figure 1). Only three positive pools originated from the site at Wagbachniederung in 2009. All three sites are located in the north of the state of Baden-Württemberg (see also below).

*Setaria tundra *appears to be more widespread: it was found in various mosquito species in the states of Bavaria, Rhineland-Palatinate and Baden-Württemberg.

### Potential vertebrate hosts and vectors

At this stage, we can only speculate about the potential vertebrate hosts of the two different filariae. In the case of *Setaria tundra *it seems prudent to assume roe-deer as the vertebrate host, as in the past this has been reported from Germany [21,37] and neighbouring countries [38-40]. In southern Germany the prevalence of *S. tundra *in roe-deers ranges between 1.6% in North Rhine-Westphalia [37] and 12.3% in northern Bavaria [21]. In Finland, roe-deer seem to be the main reservoir, but reindeer and moose can also be infected [41]. The transmission of *S. tundra *by various German mosquito species such as *Oc. sticticus*, *Ae. vexans *and *An. claviger *is similar to findings from Finland, where *Ae. communis, Ae. punctor, Ae. hexodontus and Ae. excrucians *were incriminated as natural vectors [28]. The veterinary importance of *S. tundra *is due to a reported outbreak of peritonitis with significant economic losses in Finnish semi-domesticated reindeer in 2003-5 [42]. Similar outbreaks may occur in any other wild or semi-domesticated cervid population outside Finland, e.g. in zoos.

Regarding the filarial species derived from Culex mosquitoes, birds might serve as vertebrate hosts, because of the ornithophilic behavior of most Culex species in Europe [38]. Potential bird parasite genera known from Europe are Cardiofilaria, Chandlerella, *Eufilaria*, *Eulimdana*, *Pelecitus*, *Pseudlemdana*, *Sarconema *and *Splendidofilaria *[12,43-49], some of which are found in common birds such as Blackbirds (*Turdus merula*), Magpies (*Pica pica*) or Great Tits (*Parus major*) ([12,50]), others in domesticated exotic birds [51]. However, only certain Cardiofilaria and Pelecitus species are known to be vectored by culicines from Asia and North America [12,52]. A single record from German birds of prey [47] makes *Cardiofilaria pavlovskyi *the favourite candidate for our unidentified specimens. It is also known from various birds in Spain, France, Poland and Latvia [12,45,49,50]. Final conclusions are prevented by the paradox that on one hand, sequences are not available for *Cardiofilaria *and for the majority of the about 90 known genera of onchocercid filariae [17], of which only 20 genera are currently represented in GenBank. On the other hand our study protocol did not allow morphological examination prior to homogenization for DNA extraction, and the same applies to most if not all vector-borne specimens listed in GenBank. The only bird parasite that was confirmed by morphology and DNA sequencing during a bird die-off in late 2011 was *Diplotriaena *sp. (Nematoda: Diplotriaenidae) in two dead blackcaps (*Sylvia atricapilla*), but the 12S DNA sequence turned out to be very different from the *Culex *filariae (unpublished observations by the authors).

It is generally assumed that avian filarioid parasites are rarely pathogenic, and clinical signs may occur only in some bird species or in some individuals within one species [53].

## Conclusions

This study searched for filarial human pathogens in German mosquitoes, for instance *Dirofilaria *species. In about 83,000 mosquitoes tested, no filarial DNA sequences were found that matched those known for the two potential targets, i.e. *D. immitis *or *D. repens*. Hence it remains open whether one or both parasites could be transmitted autochthonously by indigenous mosquitoes. The absence of *Dirofilaria *spp. or other zoonotic filariae in our sample allows the conclusion that the risk of autochthonous infection in Germany is still very low, although dirofilariasis is emerging and spreading in Europe [10].

The generalized filarial PCR primers used in our study allowed the detection of two other species one of which could be unambiguously identified as *Setaria tundra*. The other remains unidentified, but is likely a bird parasite.

We do not have any published information regarding human infection with *Setaria tundra *or one or the other bird filariae, but it could be possible occasionally since some of the mosquito species found to be infected are known to feed on humans. In this case, infections are likely to be symptomless, given the lack of any case report. In summary, the synergism between entomologists, virologists and parasitologists, combined with state-of-the-art methods allows a very efficient near-real-time monitoring of a wide spectrum of both human and veterinary pathogens, including the discovery of yet unknown or neglected species.

## List of abbreviations used

Bp: Base pair; COI: Cytochrome oxidase I gene; EVS: Encephalitis vector survey trap; GT: Gravid trap; MIR: Minimum infection rate; mtDNA: Mitochondrial DNA; rRNA: Ribosomal DNA.

## Competing interests

The authors declare that they have no competing interests.

## Authors' contributions

CC: As part of her doctoral thesis she conducted a significant part of the 2010 mosquito collection field-work, executed all PCRs, analysed the data and contributed to the manuscript drafting. NB coordinated most of the field-work and gave significant input to the study design and execution. SP established the conventional filaria PCR diagnostics and contributed substantially to the manuscript drafting. HJ: As part of her doctoral thesis she conducted all 2009 and a big part of the 2010 field-work, did most DNA extractions in conjunction with the arbovirus project and was involved in the data analysis and manuscript drafting. JS-C supervised the lab work, designed the filarial real-time PCR and contributed to the manuscript drafting. AK contributed to the study design and wrote the manuscript. All authors read and approved the final version of the manuscript

## Authors' information

CC is a doctoral student of Biology at the University of Heidelberg.

NB is assistant professor at the University of Heidelberg, the coordinator of the German arbovirus surveillance and mosquito monitoring program and managing director of the KABS. SP is clinical microbiologist and parasitologist at the Bernhard-Nocht-Institute for Tropical Medicine. HJ is a doctoral student of Biology at the University of Heidelberg. JS-C is assistant professor of virology at the University of Hamburg, and head of the arbovirus lab group at the Bernhard-Nocht-Institute for Tropical Medicine. AK is assistant professor of entomology at the University of Hamburg, and head of the Bundeswehr lab group for medical entomology at the Bernhard-Nocht-Institute for Tropical Medicine.
